# Achieving equity in HIV-treatment outcomes: can social protection improve adolescent ART-adherence in South Africa?

**DOI:** 10.1080/09540121.2016.1179008

**Published:** 2016-07-08

**Authors:** L. D. Cluver, E. Toska, F. M. Orkin, F. Meinck, R. Hodes, A. R. Yakubovich, L. Sherr

**Affiliations:** ^a^ Centre for Evidence-Based Intervention, Department of Social Policy and Intervention, University of Oxford, Oxford, UK; ^b^ Department of Psychiatry and Mental Health, University of Cape Town, Cape Town, South Africa; ^c^ AIDS and Society Research Unit, Centre for Social Science Research, University of Cape Town, Cape Town, South Africa; ^d^ DPHRU, School of Clinical Medicine, and DST-NRF Centre of Excellence in Human Development, University of the Witwatersrand, Johannesburg, South Africa; ^e^ Health Psychology Unit, Research Department of Infection & Population Health, University College London, London, UK

**Keywords:** Adolescents, antiretroviral therapy (ART), adherence, social protection

## Abstract

Low ART-adherence amongst adolescents is associated with morbidity, mortality and onward HIV transmission. Reviews find no effective adolescent adherence-promoting interventions. Social protection has demonstrated benefits for adolescents, and could potentially improve ART-adherence. This study examines associations of 10 social protection provisions with adherence in a large community-based sample of HIV-positive adolescents. All 10–19-year-olds ever ART-initiated in 53 government healthcare facilities in a health district of South Africa’s Eastern Cape were traced and interviewed in 2014–2015 (*n* = 1175 eligible). About 90% of the eligible sample was included (*n* = 1059). Social protection provisions were “cash/cash in kind”: government cash transfers, food security, school fees/materials, school feeding, clothing; and “care”: HIV support group, sports groups, choir/art groups, positive parenting and parental supervision/monitoring. Analyses used multivariate regression, interaction and marginal effects models in SPSS and STATA, controlling for socio-demographic, HIV and healthcare-related covariates. Findings showed 36% self-reported past-week ART non-adherence (<95%). Non-adherence was associated with increased opportunistic infections (*p* = .005, B .269, SD .09), and increased likelihood of detectable viral load at last test (>75 copies/ml) (aOR 1.98, CI 1.1–3.45). Independent of covariates, three social protection provisions were associated with reduced non-adherence: food provision (aOR .57, CI .42–.76, *p* < .001); HIV support group attendance (aOR .60, CI .40–.91, *p* < .02), and high parental/caregiver supervision (aOR .56, CI .43–.73, *p* < .001). Combination social protection showed additive benefits. With no social protection, non-adherence was 54%, with any one protection 39–41%, with any two social protections, 27–28% and with all three social protections, 18%. These results demonstrate that social protection provisions, particularly combinations of “cash plus care”, may improve adolescent adherence. Through this they have potential to improve survival and wellbeing, to prevent HIV transmission, and to advance treatment equity for HIV-positive adolescents.

## Introduction

The scale-up of HIV-treatment provides an opportunity for the survival and long-term well-being of the 1.6 million HIV-positive adolescents in Southern Africa (UNAIDS, 2015b). But studies show that adolescents are the lowest-adherent age group (Hudelson & Cluver, 2015; Nachega et al., 2009), leading to morbidity, mortality, viral resistance and onward HIV transmission (Pillay, 2001; Sherr et al., 2010). AIDS is currently the single greatest cause of death amongst adolescents aged 10–19 in Africa (UNAIDS, 2015a).

Our understanding of why adherence is so difficult for adolescents remains incomplete (Lowenthal, Bakeera-Kitaka, et al., 2014). Studies in the USA and Southern Africa find associations between non-adherence to sexual risk behaviour and mental health distress (Lowenthal et al., 2012; Mellins et al., 2011). Adolescence is a period of social, familial and emotional change. Adolescence is also a transitional period in health provisions and practices (Ferrand et al., 2010), as adolescents may move from paediatric to adolescent services, changing from caregiver-mediated to autonomous adherence.

Three recent systematic reviews find virtually no evidence-based interventions to improve adolescent adherence or retention in care (Hudelson & Cluver, 2015; MacPherson et al., 2015; Vreeman, Wiehe, Pearce, & Nyandiko, 2008). Findings from adult HIV treatment suggest that economic support may be important. This includes grants usually labelled as “cash” (now with very high coverage for children in South Africa due to successful scale-up since 2008 (Hall, 2015)) or other “cash in kind” social protections, such as clinic transport subsidies (Emenyonu et al., 2012) and food provision (de Pee, Grede, Mehra, & Bloem, 2014). Child development literature suggests that psychosocial “care” social protection from caregivers or other adults is important for healthy adolescent behaviours (Bronfenbrenner, 1979; Rutter, 2007). Regional evidence shows positive effects of social protection on other adolescent outcomes, such as sexual risk behaviours (Baird, Garfein, McIntosh, & Ozler, 2012; Cluver, Orkin, Boyes, & Sherr, 2014; DSD, SASSA, & UNICEF, 2012; Handa, Halpern, Pettifor, & Thirumurthy, 2014), mental health distress and family relationships (Bhana et al., 2014; Kilburn, Thirumurthy, Halpern, Pettifor, & Handa, 2016). Growing evidence of associations between social protection and HIV-risk reduction (Cluver et al., 2015; Pettifor, Rosenberg, & Bekker, 2016) is reflected in a number of policy documents by UNICEF, UNAIDS and PEPFAR-USAID that focus on paediatric and adolescent HIV-prevention (PEFPAR, 2015; UNAIDS, 2014; UNICEF, 2012).

To date, no known studies have examined associations of social protection (narrowly or broadly defined) with adolescent ART-adherence. Nor does any research examine whether combinations of different types of social protection may be more effective than individual provisions. “Cash” social protections that may be particularly valuable for HIV-positive adolescents include government cash transfers to households, or “cash in kind” (e.g., food to facilitate medication taking or free schooling to obviate resource spending on education). Potential “care” social protections include those provided by clinics and communities: HIV support groups (Grimsrud, Lesosky, Kalombo, Bekker, & Myer, 2016), recreational or sports groups. At home, different parenting “care” approaches may potentially support adherence, including positive parenting (praise and support) and parental supervision/monitoring of adolescent activities.

This study asks: (i) Are various forms of cash and/or care social protection provisions associated with adolescent ART-adherence? and (ii) Can combinations of social protection provisions have additive associations with adherence?

## Methods

One thousand fifty-nine ART-initiated adolescents were interviewed using clinic sampling with community tracing in a mixed urban, peri-urban and rural health district of the Eastern Cape, South Africa. From 2014–2015, all public health facilities that provided ART to >4 adolescents were identified (*n* = 53). Within each facility, all adolescents aged 10–19 who had ever initiated ART were identified through paper and computerised records. All adolescents were followed up in their homes or met at clinics, to ensure inclusion regardless of clinic attendance rates or being lost to follow-up. About 90.1% of the eligible sample was interviewed. Of the remainder, 4.1% refused participation (either adolescent or caregiver), 0.9% had such severe cognitive disability that they were unable to participate, 1.2% were unable to be interviewed for safety reasons and 3.7% were unable to be traced.

Voluntary informed consent was obtained from caregivers and adolescents for a 90-minute interview. No incentives were provided, but all adolescents were given a certificate, snack, toothbrush and toothpaste. To prevent identification or stigmatisation through HIV-related research, the study was presented locally as focusing on general needs of adolescents using social and health services. Also with this aim, 467 additional adolescents who were co-resident, or who lived in neighbouring homes, were also interviewed with a version of the questionnaire that did not include items on HIV-medication or HIV-illness (not included in these analyses).

Questionnaires, interview schedules and consent forms were translated and back-translated between English and Xhosa, and used tablets with youth-friendly graphics and interactive games. Adolescents participated in the language of their choice. Interviewers were trained in working with HIV-affected adolescents. Confidentiality was upheld, except in cases of significant harm or when participants requested assistance. Where participants reported recent abuse, rape or risk of significant harm, referrals were made to child protection and health services, with follow-up support. Ethics protocols were approved by the Universities of Cape Town (CSSR 2013/14) and Oxford (SSD/CUREC2/12-21), the Provincial Departments of Health and Education and ethics review committees of participating hospitals.

The study design was developed in collaboration with the South African National Departments of Health, Social Development and Education, UNICEF South Africa, Regional and New York Pediatric HIV teams, PEPFAR-USAID, and NGOs including Pediatric AIDS Treatment for Africa (PATA) and the Regional Psychosocial Support Initiative (REPSSI). Research tools were informed by in-depth qualitative research, and pre-piloted with 25 HIV+ adolescents in the Eastern Cape. Questionnaires, accompanying vignettes, pictures and games were developed in consultation with two Teen Advisory Groups of HIV-infected and affected adolescents from urban and rural areas of the Eastern Cape (*n* = 20) and Western Cape (*n* = 18).

### Measures

*ART adherence* was measured by adolescent self-report (Evans et al., 2015), using the standardised Patient Medication Adherence Questionnaire (Duong et al., 2001), combined with adolescent adherence measures developed in Botswana (Lowenthal, Haruna, et al., 2014). After piloting, and in order to reduce social desirability bias, vignettes were added, for example, “Even if Andiwe tries his best sometimes unexpected things get in the way and prevent him from taking his pills … this is not his fault”. Past-week and past-year non-adherence were measured using a 95% adherence cut-off, based on the number of prescribed daily doses (Paterson et al., 2000), but past-week adherence was used for all analyses due to evidence of increased reliability for more recent recall. Two validation measures of self-reported adherence were included. *Opportunistic infections* were measured as sores on the body or face, tuberculosis symptoms (e.g., coughing blood and night sweats), shingles and mouth ulcers in the past six months, using a verbal symptom checklist (Lopman et al., 2006), validated in previous studies of adults in South Africa. Additionally, for a 25% subset of adolescents from randomly selected clinics, *viral load measures* were collected from clinic files.

*Social protection provisions* included economic “cash” and psychosocial “care” provisions. Within “cash”, cash transfers were any government welfare grant provided to the household (child support, foster child, care dependency, pension or disability grant); food security was measured using items from the National Food Consumption Survey and defined as two meals daily for the past week; school access was capacity to pay for or free access to school, textbooks and uniform. School feeding was measured as daily free provision of a meal at school. Access to sufficient clothing was measured using items from the SA Social Attitudes Survey (Pillay, Roberts, & Rule, 2006). Within “care”: access to an HIV-support group was past-month attendance at either a youth-focused or general HIV-support group; access to sports, choir or arts groups was attending past-month extra-curricular organised activities. Positive parenting (i.e., praise and positive reinforcement from any primary caregiver) and parental supervision/monitoring (i.e., primary caregiver’s monitoring of adolescent activities, rules about going out) were measured using adolescent-reported subscales of the Alabama Parenting Questionnaire (Elgar, Waschbusch, Dadds, & Sigvaldason, 2007). “Parenting’ referred to any biological or non-biological primary caregiver.

*Potential covariates* that were controlled for in analyses were socio-economic factors of adolescent age, gender, language, formal/informal (shack) housing, urban/rural location and education level (highest school grade passed) measured using items adapted from the South African census (SSA, 2011). Maternal and paternal death were asked using items from a South African national survey of AIDS-affected children (Cluver et al., 2013). HIV and medication factors included perinatal/horizontal infection, using modelling data from Southern Africa (Ferrand et al., 2009), whether the adolescent lived with a caregiver who was AIDS-symptomatic or on ART, whether the adolescent was aware of their own HIV-positive status (using clinic file data and adolescent report) and duration of time on treatment using patient file data, supported by caregiver report and cross-checked with adolescent self-report. Healthcare factors included general past-month self-reported health and time of travel to clinic, and whether the participant had received care in hospital for illness in the past year.

### Analyses

Analyses were conducted in four stages in SPSS 21.0 and STATA 13.1. First, known characteristics (age, gender, urban/rural location) of excluded participants were compared to those included, to check for potential differences, and subsequently descriptive statistics for outcomes, social protection variables, and covariates were calculated, and social protection provisions were excluded from analyses where a comparison group was too small for reliable analysis (Table 1). Second (Table 2), linear and logistic regressions tested associations of self-reported non-adherence, number of opportunistic infections and detectable viral load, controlling for all potential covariates.

**Table 1.** Socio-demographic, health and social protection factors for HIV-positive adolescents (*n* = 1059).

Comparison of included and excluded participants				
	HIV+ (*n* = 1060)	Excluded (*n* = 116)	Sig.	
Age (mean, SD)	13.8, 2.834	14.8, 2.91	*z *= 1.96, *p *= .671	
Female (*n*, %)	587, 55.2	66, 56.9	*χ*^2^(df)* *= .098(1), *p *= .769	
Rural (*n*, %)	228, 21.4	26, 2.2	*χ*^2^(df)* *= .050(1), *p *= .813	
Included participants: descriptive analyses				

Factor grouping	Factor	Category	*n* (%)	Mean (SD)
HIV-related outcomes	Adherence	Past-week non-adherence	385 (36.4)	
		Past-year non-adherence	554 (52.3)	
	Opportunistic infections	Number of OIs: sores, TB, shingles and mouth ulcers		1.7 (1.4)
	Most recent viral load (*n* = 201)	Detectable >75 ml copies	120 (45.1)	
Socio-demographic factors	Age	Age in years		13.8 (2.8)
		10–14 years old	659 (62.2)	
		15–19 years old	400 (37.8)	
	Gender	Female	583 (55.1)	
		Male	476 (44.9)	
	Language	Xhosa	1027 (97.0)	
	Housing	Informal shacks/settlements	198 (18.7)	
		Formal	860 (81.3)	
	Location	Urban	828 (78.2)	
		Rural	227 (21.4)	
	Education level	Highest grade completed		5.8 (2.6)
	Orphanhood	Maternal orphan	463 (43.7)	
		Paternal orphan	319 (30.1)	
Health, HIV and medication-related factors	Mode of HIV infection	Perinatal infection	708 (66.9)	
		Horizontal infection	351 (33.1)	
	Caregiver AIDS-related characteristics	Carer AIDS-symptomatic	45 (4.2)	
		Carer taking ART	215 (20.3)	
	Knowledge of HIV status	Adolescent knows they are HIV positive	793 (74.9)	
	Access to antiretroviral medicine	Time on treatment (years)		5.9 (4.5)
	Health-specific factors	Poor past-month health status	625 (59.0)	
		Travel to clinic more than one hour	120 (11.3)	
		Recent hospital visit for illness	542 (51.2)	
Social protection provisions	Cash	Any government welfare grant	1,002 (94.7)	
		Food security (past week 2 meals/day)	820 (77.4)	
		School feeding scheme	985 (93.0)	
		School access	487 (46.0)	
		Access to clothing	863 (81.5)	
	Care	HIV support group	141 (13.3)	
		Sports group	460 (43.6)	
		Choir/art group	156 (14.7)	
		Positive parenting	442 (41.7)	
		Parental supervision/monitoring	432 (40.8)

**Table 2:** Associations of past-week self-reported non-adherence.

A. Number of opportunistic symptoms (*n* = 1059)^a^			
Covariates	*B*	SE	Beta
Age (years)	.020	.032	.041
Female gender (Y/N)	.025	.090	.009
Xhosa language (Y/N)	.362	.254	.046
Informal housing (Y/N)	.170	.117	.049
Rural location (Y/N)	–.051	.112	–.015
Highest grade completed	–.063	.029	–.119*
Maternal orphan (Y/N)	.100	.097	.037
Paternal orphan (Y/N)	–.035	.097	–.012
Perinatal infection (Y/N)	.004	.164	.001
Caregiver HIV-sickness (Y/N)	.456	.219	.071*
Caregiver on ARVs (Y/N)	–.025	.119	–.008
Knows own HIV-positive status (Y/N)	.114	.117	.035
Time on treatment (years)	–.028	.013	–.094*
Clinic travel time >1 hour (Y/N)	.341	.138	.082*
Past-month poor health (Y/N)	.685	.188	.118***
Recent hospital visit for illness	–.020	.092	–.007
Potential associated factor			
Past-week self-reported non-adherence (Y/N)	.269	.095	.093**

***p < .001, **p < .005, *p < .05.			
^a^All variables shown are entered simultaneously.			
B. Detectable viral load (>75 ml copies) (*n* = 266)^a^			
Covariates	aOR	95% CI	
Age	1.123	.926–1.362	
Female gender (Y/N)	1.056	.599–1.859	
Xhosa language (Y/N)	.887	.242–3.241	
Informal housing (Y/N)	1.178	.567–2.446	
Rural location (Y/N)	1.062	.520–2.168	
Highest grade completed	.925	.758–1.127	
Maternal orphan (Y/N)	1.199	.660–2.177	
Paternal orphan (Y/N)	1.585	.880–2.854	
Perinatal infection (Y/N)	2.668*	1.059–6.722	
Caregiver HIV-sickness (Y/N)	1.103	.303–4.016	
Caregiver on ARVs (Y/N)	1.683	.842–3.365	
Knows own HIV-positive status (Y/N)	1.783	.906–3.510	
Time on treatment (years)	.974	.896–1.058	
Clinic travel time >1 hr (Y/N)	.775	.285–2.107	
Past-month poor health (Y/N)	2.351	.726–7.617	
Recent hospital visit for illness	.939	.534–1.651	
Potential associated factor			
Past-week self-reported non-adherence (Y/N)	1.976*	1.131–3.450	

***p < .001, **p < .005, *p < .05.

^a^All variables shown are entered simultaneously.

Third (Table 3), associations between specific social protection provisions and past-week ART non-adherence were assessed, following the sequential approach recommended by Hosmer and Lemeshow (1989). Three logistic regression models were run: (a) with all potential covariates and potential social protection factors to control for potential confounding from non-randomised allocation of social protection provisions, (b) with all covariates and all potential social protection factors significant at .1 or below and (c) with only those covariates and social protection factors significant at .05 or below.

**Table 3.** Logistic regression of all potential social protection factors and covariates.

	Outcome: Past-week self-reported non-adherence		
	OR	95% CI	*p*-Value
**Stage 1: All potential covariates and social protection factors**			
Age (years)	1.052	.946–1.170	.349
Female gender	1.133	.826–1.554	.438
Xhosa language (Y/N)	2.430^a^	.875–6.748	.088
Informal housing (Y/N)	.775	.525–1.144	.199
Rural location (Y/N)	1.249	.866–1.802	.234
Highest grade completed	.972	.881–1.071	.564
Maternal orphan (Y/N)	1.002	.728–1.381	.989
Paternal orphan (Y/N)	1.123	.816–1.546	.475
Perinatal Infection (Y/N)	.951	.556–1.627	.856
Caregiver AIDS-sickness (Y/N)	1.082	.541–2.163	.823
Caregiver on ARVs (Y/N)	1.216	.828–1.785	.318
Knows own HIV-positive status (Y/N)	.711^a^	0482–1.049	.085
Time on treatment (years)	1.000	.958–1.043	.999
Clinic travel time >1 hour (Y/N)	1.668*	1.082–2.572	.020
Past-month poor health (Y/N)	1.346	.745–2.432	.325
Recent hospital visit for illness (Y/N)	.626**	.463–.846	.002
Cash – Food security (Y/N)	.668*	.463–.966	.032
Cash – School access (Y/N)	1.019	.750–1.384	.905
Cash – Clothing access (Y/N)	.843	.565–1.259	.405
Care – HIV support group (Y/N)	.682^a^	.431–1.077	.100
Care – Sport group (Y/N)	1.370^a^	.991–1.894	.057
Care – Choir/ arts group (Y/N)	1.061	.697–1.617	.782
Care – Positive parenting (Y/N)	1.043	.767–1.419	.786
Care – Parental supervision/monitoring (Y/N)	.568***	.411–.785	.001
**Stage 2: All covariates and social protection significant <.1**			
Xhosa language (Y/N)	1.803	.775–4.199	.171
Clinic travel time >1 hour (Y/N)	1.387	.929–2.071	.110
Recent hospital visit for illness (Y/N)	.542***	.416–.705	<.001
Knows own HIV-positive status	.772	.568–1.050	.099
Cash – Food security (Y/N)	.551***	.407–.746	<.001
Care – HIV support group (Y/N)	.636*	.417–.970	.035
Care – Sport group (Y/N)	1.140	.874–1.486	.333
Care – Good parental supervision/monitoring (Y/N)	.524***	.398–.690	<.001
**Stage 3: All covariates and social protections significant <.05**			
Recent hospital visit for illness (Y/N)	.552***	.426–.716	<.001
Cash – Food security (Y/N)	.565***	.418–.763	<.001
Care – HIV support group (Y/N)	.603*	.401–.906	.015
Care – Good parental supervision/monitoring (Y/N)	.557***	.426–.728	<.001

Note: All variables entered simultaneously in each stage.

***p < .001, **p < .005, *p < .05, ^a^ *P* < .10.

**Figure 1.**
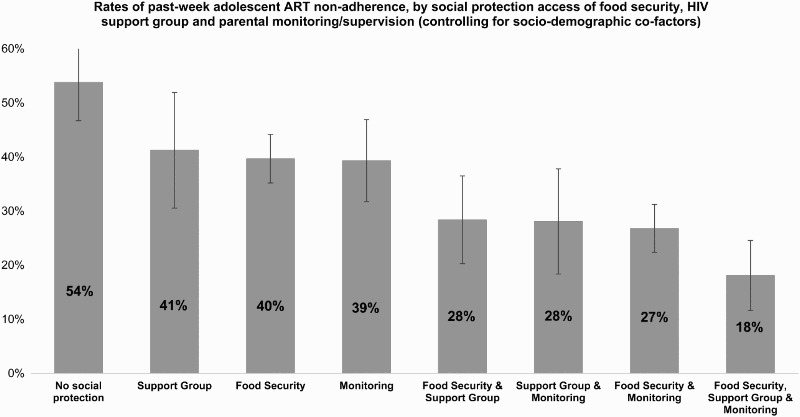
Marginal effects model testing for additive effects of combination social protections on adolescent ART-adherence.

Fourth, we tested for potential interactive or additive effects on adolescent ART-adherence of combinations of social protections. To test for interactive effects, a logistic regression included all covariates, significant social protection provisions (using only those significant in Stage 3 above), and all possible two-way and three-way interactions of significant social protections. To identify potential additive effects, all potential combinations of the statistically significant social protection variables were entered into a marginal effects analysis using binary logistic regression, with covariates held at their mean values. This analysis indicated how the predicted probability of the outcome changed when different interventions (and combinations of interventions) were present (Figure 1).

## Results

### Descriptives (Table 1)

#### Outcomes

There were no significant differences between included and excluded participants on known factors of age, gender and urban/rural location. About 36% of adolescents reported ART non-adherence in the past week, and 52% reported non-adherence in the past year. Past-week reporting was used for all further analyses. Adolescents reported a mean of 1.7 current opportunistic infections (median 2.0, SD 1.4, range 0–5). Within the subset of adolescents where we collected viral load measures (*n* = 266), 45.1% had a detectable viral load (>75 copies/ml).

#### Socio-demographic, HIV and healthcare covariates

The sample had a mean age of 13.8 (median 13.0, SD 2.8, range 10–19), was 55% female, and 97% first-language Xhosa; 19% lived in informal housing and 81% in formal homes, with 21% in rural areas and the remainder in peri-urban or urban locations. On average, adolescents reported completing nearly 6 grades of school (mean 5.77, SD 2.6, median 6.0, range 0–12); 44% were maternally and 30% paternally orphaned, with a further 3% of mothers and 16% of fathers non-resident; 67% were perinatally infected. Adolescents had been on ART for a mean of 5.9 years (median 5.0, SD 4.5, range 0–19 years), and 59.9% reported poor/not good health in the past 6 months. Mean travel time to healthcare facilities was three quarters of an hour (median 30 minutes, SD 95 minutes, range 1 minute to 3 hours).

#### Social protection access

In all, 95% of adolescents received a cash transfer grant in their household, and 93% had regular school feeding; 78% had enough food to eat in the past week, 46% had access to school, uniform and textbooks; 81% had enough clothes to stay warm and dry, 13% attended any HIV support group, 13% were part of a sports team or organised sports group and 15% attended a choir or arts group. Forty-two per cent reported high positive parenting and 41% reported high parental supervision.

### Validating self-reported adherence (Table 2)

Regression analyses controlling simultaneously for all potential covariates (age, gender, language, housing type, location, education, maternal/paternal orphanhood, perinatal infection, caregiver AIDS-illness, caregiver ART-taking, status knowledge, time on treatment, clinic travel time, health status and hospitalisation) showed that past-week non-adherence was associated with increased rates of current opportunistic infections (*B* 0.269, *p* < .006), with adolescents reporting a mean of 1.7 OIs (SD 1.36, range 0–5) in the past six months. In the 25% subsample (*n* = 266), past-week non-adherence was significantly associated with increased rates of detectable viral load (aOR 1.98, CI 1.13–4.45, *p* < .05). Median viral load of adolescents with detectable VL was 689 copies/ml.

### Independent associations of social protection provisions with non-adherence (Table 3)

Associations of eight potential social protection provisions with ART non-adherence were tested simultaneously (government cash transfers and school feeding were excluded due to less than 100 adolescents not receiving the provision), controlling for all the potential covariates (age, gender, language, housing type, location, education, maternal/paternal orphanhood, perinatal infection, caregiver AIDS-illness, caregiver ART-taking, status knowledge, time on treatment, clinic travel time, health status and hospitalisation, all entered simultaneously). Table 3 shows the three factors that remained significantly associated with reduced non-adherence, independent of all other social protection factors and covariates. Sufficient food (aOR 0.57, CI 0.42–0.76, *p* < .001); attending an HIV support group (aOR 0.60, CI 0.40–0.91, *p* < .05) and high parental supervision/monitoring (aOR 0.56, CI 0.43–0.73, *p* < .001) were associated with reduced non-adherence.

### Associations of combination social protection interventions with non-adherence

Using only the three social protection provisions significantly associated with adherence, potential multiplicative effects of two-way and three-way combinations (food × support group, supervision × support group, food × supervision, food × support group × supervision) were tested using interaction terms in logistic regression, controlling for all covariates. No statistically significant interactions were shown, indicating no multiplicative effects (Table 4). Consequently, to investigate potential additive effects of the three social protections that were all significant independently of each other, we calculated interval estimates of the predicted probability of the outcome when different combinations of social protections are received, whilst controlling for covariates. We found strong additive effects of combining social protection provisions. Predicted probability of past-week non-adherence for adolescents not receiving sufficient food, support group or good parental monitoring was 54%. With any one of these social protections, predicted probability of past-week non-adherence was 39–41%, and with any two, 27–28%. With all three social protection provisions, predicted probability of past-week non-adherence was 18% (Figure 1).

**Table 4.** Logistic regression of all significant potential social protection factors, interaction terms and covariates.

	Outcome: Past-week self-reported non-adherence		
	OR	95% CI	*p*-Value
**Main effects**			
Recent hospital visit for illness (Y/N)	.552***	.426–.716	<.001
Cash – Food security (Y/N)	.565***	.418–.763	.003
Care – HIV support group (Y/N)	.603**	.401–.906	<.001
Care – Parental supervision/monitoring (Y/N)	.557***	.426–.728	<.001
**Conditional and interaction effects**			
Recent hospital visit for illness (Y/N)	.555***	.427–.720	<.001
Cash – Food security (Y/N)	.548**	.365–.825	.007
Care – HIV support group (Y/N)	.620	.262–1.470	.020
Care – Parental supervision/monitoring (Y/N)	.492*	.274–.833	.001
Interaction – Food security BY HIV support group	.937	.332–2.643	.727
Interaction –Parental supervision BY HIV support group	1.619	.282–9.288	.567
Interaction – Parental supervision BY Food security	1.168	.599–2.278	.216
Interaction – Food security BY HIV support group BY Parental supervision	.568	.073–4.430	.481

***p < .001, **p < .005, *p < .05.

## Discussion

Findings have three key messages for approaches to treatment rollout in Southern Africa. First, that HIV-positive adolescents are at high risk of ART non-adherence, with no associations of socio-demographic factors such as age, gender and mode of infection. Self-reported data show that more than a third of adolescents were non-adherent in the past week, and half were non-adherent in the past year. Associations of non-adherence with more opportunistic infections and higher viral loads have direct clinical implications for adolescent survival. This is an immediate and urgent challenge.

Second, findings show that specific social protection provisions, “cash plus care”, are associated with reduced non-adherence. Daily provision of at least two meals per day, attending an HIV-support group, and high levels of parental supervision/monitoring may facilitate ART adherence among HIV-positive adolescents. These findings should act as an impetus for programming, as we have evidence-based programmes for all these provisions: feeding programmes, HIV-support groups and parenting interventions (Grimwood et al., 2012; Knerr, Gardner, & Cluver, 2013; Snyder et al., 2014). Scaling up such programming and ensuring access for HIV-positive adolescents are essential.

Third, data show that combinations of social protection provisions are associated with greater reductions in non-adherence than single provisions alone. The combination of cash and care social protections was associated with reductions in past-week non-adherence from 54% with none of these social protections to 18% with all three. It may be that multi-component interventions are more beneficial in combatting the complex challenges that HIV-positive adolescents face simultaneously across different parts of their lives.

This study has a number of limitations. First, data are cross-sectional and causality cannot be determined in non-randomised designs. Inclusion of a wide range of pre-selected covariates in all analyses aims to mitigate the non-randomised allocation of provisions, but these identified programmatic options should be tested in future longitudinal data and randomised trials.

Second, this study aimed to include a real-world sample of adolescents initiated by government healthcare facilities in a low-resource area of Southern Africa – reflecting the majority of the world’s population of ART-initiated adolescents. The sample reached over 90% of eligible adolescents, and rates of perinatal/horizontal transmission parallel recent estimates of the overall HIV+ adolescent population (Stover, 2014). The small groups of refusals, untraceable adolescents and adolescents with very severe cognitive delay may represent particularly high-risk subsamples for future research. However, this study is unique in being the only known study to include both clinic-attending and non-attending adolescents, by following up all ART-initiated participants to their homes, and inclusion rates were very high for such a vulnerable group.

Third, it is important to test these findings in other contexts. The study’s location was selected by state and bilateral partners as most closely reflecting poverty and healthcare contexts in other low-income Southern African contexts. The Eastern Cape is South Africa’s poorest province, with 30% antenatal HIV-prevalence, and health provision impacted by historical and current resource factors (National Department of Health, 2013).

Fourth, South Africa’s highly successful national scale-up of government cash transfers for adolescents resulted in such high coverage in this sample that government grants were almost universally received and thus not utilised in these analyses. However, economic support encompasses more than cash grants alone, and our data show that wider material provision in the form of food, clothing and school fees can be considered in the category. A broad definition of financial support is vital. Investigation of associations of cash transfers and adherence in countries with lower cash grant coverage will be essential to identify potential effects of this important social protection form on its own as well as in combination with wider financing. It will also be valuable to examine associations of social protection with ART-adherence in adolescent groups who may not have ever accessed targeted health care, including young MSM, adolescents engaged in sex work, and adolescents in institutions or living on the streets.

Finally, this study primarily uses self-reported adherence data, which risks under-reporting due to social desirability and recall bias. However, research on adherence has identified unreliability in all measurement approaches. For example, healthcare provider and parents reporting of adolescent adherence has been shown to be far higher than self-report (Evans et al., 2015). Pill counts may be manipulated by youth using techniques such as pill-dumping, and biomarkers such as viral load may be influenced by infections and viral resistance as well as ART non-adherence. In this study, validation checks against opportunistic infections and viral load measures suggest correlations between self-reported non-adherence and clinical outcomes, and support similar findings in multisite studies (Buscher, Hartman, Kallen, & Giordano, 2011; Fletcher et al., 2005; Usitalo et al., 2014).

Despite these limitations, this study has great value for informing programmatic approaches to adolescent HIV-treatment. It uses existing interventions that are currently provided by states, NGOs, or families at a large scale. It demonstrates that social protection combinations remain strongly associated with reductions in non-adherence independently of a wide range of socio-demographic, HIV-related and healthcare factors. This study takes place in real-world, low-resource contexts, with a highly representative sample of adolescents who have ever entered a government ART programme. In doing so, it is the first study of its kind. This study provides promising initial evidence that – even in these highly challenging contexts – combination social protection may have the potential to reduce non-adherence. And this in turn could contribute to reducing inequitable rates of adolescent AIDS deaths, the potential for viral resistance and treatment failure, the need for expensive and complex salvage regimes, and onward transmission of HIV.
